# Missing and Corrupted Data Recovery in Wireless Sensor Networks Based on Weighted Robust Principal Component Analysis

**DOI:** 10.3390/s22051992

**Published:** 2022-03-03

**Authors:** Jingfei He, Yunpei Li, Xiaoyue Zhang, Jianwei Li

**Affiliations:** Tianjin Key Laboratory of Electronic Materials and Devices, School of Electronics and Information Engineering, Hebei University of Technology, Tianjin 300401, China; liyunpei_hebut@163.com (Y.L.); zxy_zhangxiaoyue@163.com (X.Z.); ljw__0917@163.com (J.L.)

**Keywords:** wireless sensor networks, missing and corrupted data recovery, weighted nuclear norm, robust principal component analysis

## Abstract

Although wireless sensor networks (WSNs) have been widely used, the existence of data loss and corruption caused by poor network conditions, sensor bandwidth, and node failure during transmission greatly affects the credibility of monitoring data. To solve this problem, this paper proposes a weighted robust principal component analysis method to recover the corrupted and missing data in WSNs. By decomposing the original data into a low-rank normal data matrix and a sparse abnormal matrix, the proposed method can identify the abnormal data and avoid the influence of corruption on the reconstruction of normal data. In addition, the low-rankness is constrained by weighted nuclear norm minimization instead of the nuclear norm minimization to preserve the major data components and ensure credible reconstruction data. An alternating direction method of multipliers algorithm is further developed to solve the resultant optimization problem. Experimental results demonstrate that the proposed method outperforms many state-of-the-art methods in terms of recovery accuracy in real WSNs.

## 1. Introduction

Wireless sensor networks (WSNs) contain a group of spatially distributed sensor nodes that are capable of communicating wirelessly and collecting data from the surrounding environments [1,2]. Recently, WSNs have been widely applied in different domains, such as environmental monitoring [3], military management [4], and health care [5]. Typically, the main task of WSNs is to collect sensing data from all sensor nodes to a certain sink and then perform further analysis based on the monitoring data, and the collected data are usually composed of readings sensed by multiple nodes in consecutive time slots. However, due to the poor environments and energy constraints in WSNs, data loss and corruption are inevitable in practical applications. Therefore, it is important to reconstruct the real data from partially collected data with corruption.

Recently, various reconstruction methods have been proposed for data recovery in WSNs. Based on data interpolation techniques, a K nearest neighbor (KNN)-based method [6] was proposed to simply utilize the values of the nearest neighbors to estimate the missing values. The Delaunay triangulation (DT) [7] utilizes the vertices as their global errors to reconstruct virtual triangles for data interpolation. Based on compressed sensing (CS) [8], the distributed compressed sensing (DCS) method [9,10] was proposed to exploit the sparsity of the data under various transform domains.

Since many signals in various applications are always distributed into two-dimensional data (i.e., matrix form) and exhibit second-order sparsity (i.e., the low-rankness), matrix completion (MC) [11] has emerged as a novel technology and has been applied to many fields, such as image inpainting [12], magnetic resonance imaging [13], and recommendation systems [14]. The matrix completion aims at recovering the missing entries of a low-rank matrix from the incompletion observations, which can be formulated as a rank minimization problem. In general, solving this problem is NP-hard, since the rank function is non-convex. Fortunately, the nuclear norm, the sum of all singular values of the matrix, is the convex approximation of the rank function and can be used as an alternative [11].

Since the readings collected from N nodes during M time slots in WSNs can also be distributed into a matrix exhibiting low-rankness, the matrix completion-based methods have been proposed to utilize the correlation of WSNs data. An efficient data collection approach (EDCA) [15] and spatiotemporal compressive data collection (STCDG) method [16] were firstly proposed to recover the WSNs data by exploiting the spatiotemporal correlation in the form of low-rankness. Recently, several methods jointly utilizing low-rank and spatiotemporal sparsity feature [17,18] were proposed. Considering that the missing of row of the data matrix due to a broken node will greatly degrade the recovery accuracy, the matrix completion method [19] was proposed to utilize the interpolation technique for WSNs data recovery. In addition, in order to address the needs of real-time reconstruction of data in practical applications, the sliding window-based reconstruction approach [20,21] was proposed to achieve real-time data recovery.

However, the reconstruction performance of these methods will greatly degenerate when corruption exists in the sampled data. Direct constraint of the low-rankness cannot avoid the impact of corruption on the reconstruction of normal data. A two-phase MC-based data recovery scheme (MC-Two-Phase) [22] was proposed to recover the normal data without the influence of corruption by detecting the corruption with the principal component analysis (PCA) [23] before reconstruction. Although PCA can be utilized to detect faults corrupted by small noise, it has the problem of poor robustness. To overcome the limitations of PCA, the robust principal component analysis (RPCA) method [24,25,26] have been proposed in recent years. The RPCA method improves the robustness since it only emphasized that the noise is sparse regardless of the strength of the noise. However, it is unreasonable to treat all singular values equally in the traditional RPCA algorithm, since different singular values may contain signal information with different important levels.

To solve the above problem, we propose a weighted robust principal component analysis (WRPCA) method for the reconstruction of WSN data with corruption. The main contributions of this paper are the following:

Firstly, based on RPCA, the original data with outliers are decomposed into a sum of a low-rank normal data matrix and a sparse abnormal matrix to avoid the influence of outliers during reconstruction.

Secondly, the low-rankness of WSNs data is revealed by the variation of singular values of two real datasets collected from the Inter Berkeley Research lab and GreenOrbs.

Thirdly, the weighted nuclear norm is introduced to constrain the low-rankness and preserve the principal components of WSNs data.

The rest of this paper is organized as follows. Section 2 presents the basics of RPCA. Section 3 describes the proposed method and the reconstruction method. Section 4 shows the result of computer experiments and analysis, which is followed by the conclusion of the paper in Section 5.

## 2. Basics of RPCA

Although PCA can be used to detect corruptions, it is sensitive to gross noise and outliers. The performance and applicability of PCA are limited due to the lack of robustness to gross corruptions in real-life scenarios. As an improvement of PCA, RPCA can handle grossly corrupted data well. Suppose that data matrix X can be viewed as consisting of the two components: a low-rank matrix L and a sparse matrix S:

(1) X=L+S.

The low-rank matrix L and sparse matrix S can be obtained by solving the following problem:

(2) $$\begin{array}{c} \underset{L,S}{\min}\;\mathrm{rank}\left(L\right)+\lambda {\left\|S\right\|}_{0}\\ \;s.t.\;X=L+S, \end{array}$$

where $\mathrm{rank}\left(\cdot \right)$ denotes the rank of the matrix, ${\left\|\cdot \right\|}_{0}$ is the $l_{0}$ norm, and λ is the balance parameter.

Equation (2) is non-convex and NP-hard, which is difficult to solve. Typically, the matrix nuclear norm, the convex approximation of the rank function, can be used as an alternative. Therefore, the above problem can be cast as the following convex optimization problem:

(3) $$\begin{array}{c} \underset{L,S}{\min}\;{\left\|L\right\|}_{*}+\lambda {\left\|S\right\|}_{1}\\ s.t.\;X=L+S, \end{array}$$

where ${\left\|L\right\|}_{*}=\underset{i}{\sum }\sigma_{i}\left(L\right)$ denotes the nuclear norm of matrix L, $\sigma_{i}\left(L\right)$ is the i-th singular value of matrix L, and ${\left\|\cdot \right\|}_{1}$ is the $l_{1}$ norm. The main goal of (3) is to reconstruct low-rank normal data L from the corrupted observation data X.

RPCA has been successfully applied in different domains, including image processing [27], multimedia [28], document analysis [29], etc. The nuclear norm minimization utilized in (3) shrinks all the singular values equally [30], ignoring that different singular values may have different importance.

Actually, the real data sensed in the monitoring area always exhibit low-rankness, and the unavoidable corrupted data are sparsely distributed in the sensed data matrix. Based on RPCA, we propose a weighted robust principal component analysis method to recover the missing data in WSNs with the data corruption.

## 3. The Proposed Method

### 3.1. Problem Formulation and Signal Feature

Consider a WSN consisting of one sink and N sensor nodes, and the sensor nodes sense the environmental information and send the signal to the sink in each time slot. During M time slots, N×M readings are gathered in the sink and can be organized into a matrix $X\in {\mathbb{R}}^{N\times M}$.

However, due to hardware and network conditions, data loss and corruption may occur in the network. Mathematically, only partial data $d=\Omega \left(X\right)$ can be successfully collected in the sink, and the original data X contain the corrupted data. Here, $\Omega \left(\cdot \right)$ is the random sampling operator. That is, under the sampling ratio $\rho_{s}$, for a matrix $X\in {\mathbb{R}}^{N\times M}$, there are $d=\Omega \left(X\right)\in {\mathbb{R}}^{D\times 1}$ entries that are sampled from the whole data randomly, where $D=\left\lfloor \rho_{s}NM+\frac{1}{2}\right\rfloor$. It is worth noting that the sampled partial data d also contains the sampled corruption data. It is necessary to reconstruct the uncorrupted whole data from the sampled partial data under the sampling ratio $\rho_{s}$.

The data sensed in a certain area during a consecutive time are always redundant and highly correlated and can be distributed into a matrix (uncorrupted matrix L) exhibiting low-rankness. Since the outliers in real WSN are uniformly and randomly distributed, the sparsely distributed corrupted data can be denoted by the matrix S. Therefore, the whole data X can be regarded as a combination of the uncorrupted data matrix L and the corrupted matrix S.

**Figure 1:**
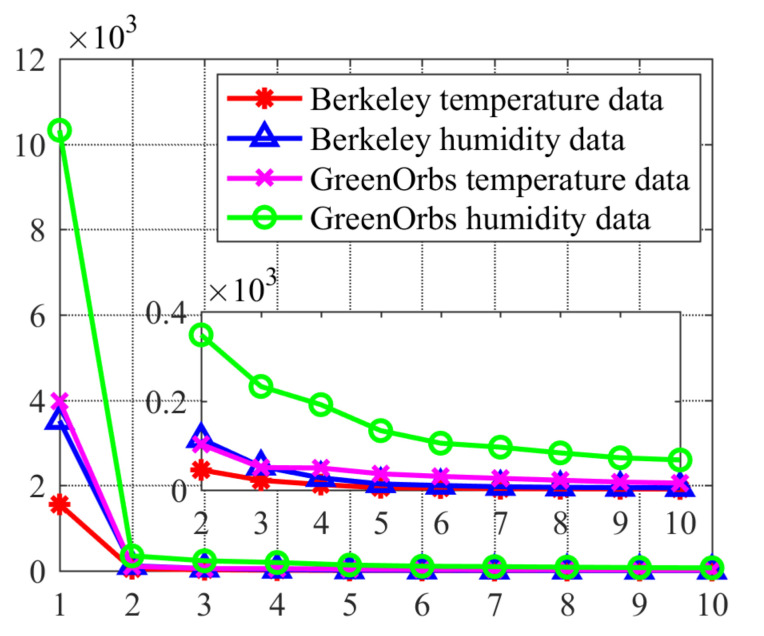
The first 10 singular values of two attribute data matrix for Berkeley and GreenOrbs data.

In order to verify that the uncorrupted data L in WSNs is low-rank, two datasets from the Inter Berkeley Research lab [31] and GreenOrbs [32] were used as testing data. Since data loss and corruption exist in both two datasets, two small but completed subset data without corruption are selected as the ground truth for our verification experiment. Specifically, the selected Inter Berkeley Research lab subset data including temperature and humidity data were measured by 49 sensor nodes during 138 time slots, and the selected GreenOrbs subset data were measured by 130 sensor nodes during 129 time slots. As shown in Figure 1, the singular values of the two attribute data matrix illustrate the low-rankness for both two datasets.

### 3.2. Proposed Method

Since the original data matrix X can be decomposed into a low-rank matrix L and a sparse matrix S, the WSNs data recovery problem can be expressed by (3). However, the NNM method adopts the same threshold for each singular value, which is not appropriate because the larger singular values usually represent the major data components of the data and contain more signal information. The larger singular values should be shrunk less to preserve the major data components.

In order to improve the practically and flexibility of the nuclear norm, the weighted nuclear norm is utilized in the recovery of WSNs data. The weighted nuclear norm of matrix L is defined as:

(4) $${\left\|L\right\|}_{w,*}=\sum_{i}w_{i}\sigma_{i}\left(L\right),$$

where $w_{i}$ is the weight coefficient, and $\sigma_{i}\left(L\right)$ is the i-th singular value of L. It is clear that the weighted nuclear norm becomes the conventional nuclear norm when $w_{1}=w_{2}=\cdots =w_{n}$.

The weighted nuclear norm minimization (WNNM) based low-rank matrix completion problem can be described as:

(5) $$L=\arg\underset{L}{\min}{\left\|Y-L\right\|}_{F}^{2}+\lambda {\left\|L\right\|}_{w,*}$$

Gu et al. [30] proved that the problem can be solved by the following singular value thresholding formula:

(6) $$L_{k+1}=shrink(Y_{k},w_{i})$$

The larger singular values should be given smaller weights to achieve less shrinkage, and the smaller ones should be given greater weights to achieve more shrinkage. The weights should be inversely proportional to singular values. Therefore, in this paper, we set the weight as:

(7) $$w_{i}=\frac{c\cdot \sqrt{M}\cdot \sigma^{2}}{\sigma_{i}\left(L\right)+\varepsilon },$$

where c>0 is a constant, M is the number of columns in L, $\sigma^{2}$ is the variance of noise, and ε only needs to be a very small number to avoid dividing by zero.

By introducing the weighted approach, different singular values are shrunk differently with weight $w_{i}$, which further preserves the major components of data. Then, a WSNs data reconstruction method is proposed by applying WNNM in traditional RPCA to recover L from partial measurement d. It can be described as:

(8) $$\begin{array}{c} \underset{L,S}{\min}\;{\left\|L\right\|}_{w,*}+\lambda {\left\|S\right\|}_{1}\\ s.t.\;\;X=L+S,\;d=\Omega \left(X\right). \end{array}$$

Only partial measurement d is known as a prior in (8). The original data X and uncorrupted L can be reconstructed from the partial data d. By introducing a quadratic penalty term, (8) can be converted to the following formulation:

(9) $$\begin{array}{c} \underset{L,S}{\min}\;{\left\|d-\Omega \left(X\right)\right\|}_{2}^{2}+\mu {\left\|L\right\|}_{w,*}+\lambda {\left\|S\right\|}_{1}\\ s.t.\;\;X=L+S, \end{array}$$

where μ and λ are the regularization parameters. The proposed method incorporates both the RPCA and WNNM in a single formulate to further preserve the major data components. The recovered $\hat{L}$ can be obtained as the uncorrupted completed data in WSNs.

### 3.3. Model Optimization

To solve (9), a reconstructed algorithm based on an alternating direction method of multipliers (ADMM) [33,34] is introduced. The augmented Lagrangian function of (9) can be written as:

(10) $$\begin{array}{cc} L\left(X,L,S,A\right)\;= & {\left\|d-\Omega \left(X\right)\right\|}_{2}^{2}+\mu {\left\|L\right\|}_{w,*}+\lambda {\left\|S\right\|}_{1}\\ & +\langle A,L+S-X\rangle +\frac{\alpha }{2}{\left\|L+S-X\right\|}_{F}^{2}, \end{array}$$

where A is the Lagrangian multiplier, and α is the penalty parameter. More details of the proposed algorithm are given as follows.

For the X-subproblem, we update $X_{k+1}$ as follows:

(11) $$\begin{array}{cc} X_{k+1} & =\arg\underset{X}{\min}L\left(X,L_{k},S_{k},A_{k}\right)\\ & =\arg\underset{X}{\min}{\left\|d-\Omega \left(X\right)\right\|}_{2}^{2}+\frac{\alpha }{2}{\left\|L_{k}+S_{k}-X+\frac{A_{k}}{\alpha }\right\|}_{F}^{2}. \end{array}$$

Here, the preconditional conjugate gradient (PCG) algorithm is applied to solve this problem in this paper.

For the L-subproblem, we update $L_{k+1}$ as follows:

(12) $$\begin{array}{cc} L_{k+1} & =\arg\underset{L}{\min}L\left(X_{k+1},L,S_{k},A_{k}\right)\\ & =\arg\underset{L}{\min}\mu {\left\|L\right\|}_{w,*}+\frac{\alpha }{2}{\left\|L+S_{k}-X_{k+1}+\frac{A_{k}}{\alpha }\right\|}_{F}^{2}. \end{array}$$

In general, the WNNM problem is non-convex. Gu et al. [30] proved that the problem has a fixed point and can be solved by the singular value thresholding formula:

(13) $$L_{k+1}=US_{w}\left(\Sigma \right)V^{T},$$

where $\left[U,\Sigma ,V\right]=\mathrm{SVD}\left(X_{k+1}-S_{k}-\frac{A_{k}}{\alpha }\right)$ is the Singular Value Decomposition (SVD) of $X_{k+1}-S_{k}-\frac{A_{k}}{\alpha }$, and $S_{w}\left(\Sigma \right)$ denotes taking singular value thresholding to the diagonal matrix Σ. Since the threshold can be effected by $w_{i}$ and μ, here, we set $w_{i}=\frac{\mu \cdot \sqrt{M}\cdot \sigma^{2}}{\sigma_{i}\left(L\right)+\varepsilon }$ to simplify the solution; then, $S_{w}{\left(\Sigma \right)}_{ii}=\max\left(\Sigma_{ii}-w_{i},0\right)$. The initial $\sigma_{i}\left(L_{k+1}\right)$ can be estimated as:

(14) $$\sigma_{i}\left(L_{k+1}\right)=\sqrt{\max\left(\Sigma_{ii}^{2}-M\sigma^{2},0\right)}.$$

For the S-subproblem, we update $S_{k+1}$ as follows:

(15) $$\begin{array}{cc} S_{k+1} & =\arg\underset{S}{\min}L\left(X_{k+1},L_{k+1},S,A_{k}\right)\\ & =\arg\underset{S}{\min}\lambda {\left\|S\right\|}_{1}+\frac{\alpha }{2}{\left\|L_{k+1}+S-X_{k+1}+\frac{A_{k}}{\alpha }\right\|}_{F}^{2}. \end{array}$$

We can find the solution via the well-known soft thresholding formula:

(16) $$S_{k+1}=soft\left(X_{k+1}-L_{k+1}-\frac{A_{k}}{\alpha },\frac{\lambda }{\alpha }\right).$$

For the A-subproblem, we update $A_{k+1}$ as follows:

(17) $$A_{k+1}=A_{k}+\alpha \left(L_{k+1}+S_{k+1}-X_{k+1}\right).$$

In practical implementation, we initialize $X_{0}$, $L_{0}$, $S_{0}$, and $A_{0}$ as the zeros matrices. Then, (9) can be solved by repeating the above steps until ${\left\|L_{k+1}-L_{k}\right\|}_{F}/{\left\|L_{k}\right\|}_{F}$ is smaller than a predefined tolerance parameter or the number of iterations reaches the predefined maximum.

The main computational cost of (9) depends on the update of $L_{k+1}$, which requires computing the SVD of the N×M matrix per iteration. The computational complexity per iteration is $O\left(\min\left\{NM^{2},\;N^{2}M\right\}\right)$.

## 4. Experiments and Analysis

Most existing WSNs data reconstruction methods (e.g., KNN [6], CS [9], EDCA [15], and methods utilizing both low-rank and sparsity feature [17,18]) have achieved satisfying recovery performances, but they do not consider the case that the WSNs data have outliers. Therefore, the performance of our proposed method is compared with the RPCA method [24] and MC-Two-Phase method [22], which consider outliers during reconstruction.

### 4.1. Experimental Environments

The two datasets adopted to verify the low-rank property of normal WSNs data were also utilized for the reconstruction experiments. The normal data without corruption can be denoted by $L_{\mathrm{nor}}$, and let $L_{\mathrm{nor}\_B}$ and $L_{\mathrm{nor}\_G}$ denote the normal matrix for Berkeley data and GreenOrbs data, respectively. The normal data can be regarded as the ground truth WSNs data. In real WSNs, due to data loss and corruption, the measurement d is partially sampled and contains corruption. To obtain the partial measurement d from normal matrix $L_{\mathrm{nor}}$ in the experiment, the following steps were performed. Firstly, the partial sampled normal data $d_{\mathrm{nor}}\in {\mathbb{R}}^{D\times 1}$ can be obtained by $d_{\mathrm{nor}}=\Omega \left(L_{\mathrm{nor}}\right)$ according to the sampling ratio $\rho_{s}$. Then, $\rho_{c}\times D$ entries in $d_{\mathrm{nor}}$ were randomly selected as the corruption data by adding additional random Gaussian noise with zero mean and variance $\sigma^{2}=20$, where $\rho_{c}$ is the corruption ratio.

The parameters λ, μ and α can be chosen according to the characteristics of the signal collected by the sink. In this paper, ε, λ, μ, and α are set to $10^{-6}$, 0.05, 3.3, and 0.05, respectively.

The Normalized Mean Absolute Error (*NMAE*) is used to measure the recovery performance of different methods on missing data and corrupted data:

(18) $$NMAE_{\mathrm{loss}}=\frac{\sum_{i,j\in \Pi_{m}}\left|L_{\mathrm{nor}}\left(i,j\right)-\hat{L}\left(i,j\right)\right|}{\sum_{i,j\in \Pi_{m}}\left|L_{\mathrm{nor}}\left(i,j\right)\right|},$$

(19) $$NMAE_{\mathrm{cor}}=\frac{\sum_{i,j\in \Pi_{c}}\left|L_{\mathrm{nor}}\left(i,j\right)-\hat{L}\left(i,j\right)\right|}{\sum_{i,j\in \Pi_{c}}\left|L_{\mathrm{nor}}\left(i,j\right)\right|},$$

where $\hat{L}$ is the recovered data, $\Pi_{m}$ denotes the missing data set, and $\Pi_{c}$ is the corrupted data set. The experimental result is an average of 50 repeated experiments.

### 4.2. Recovery Performance Comparisons

**Figure 2:**
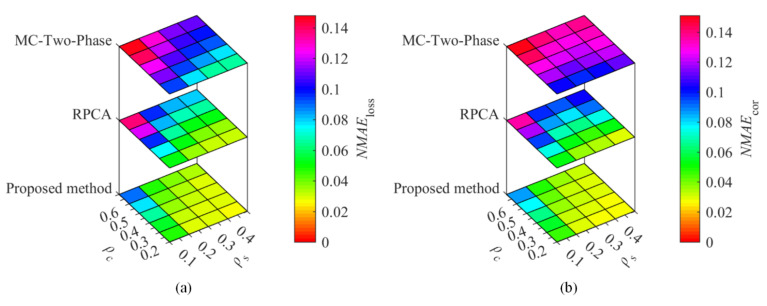
The recovery performance of each method for temperature data sensed in the Inter Berkeley Research lab. The (**a**) is the NMAE of missing data, (**b**) the NMAE of corrupted data.

**Figure 3:**
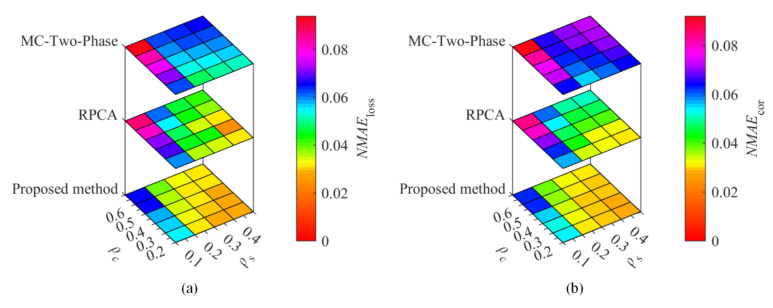
The recovery performance of each method for humidity data sensed in the Inter Berkeley Research lab. The (**a**) is the NMAE of missing data, (**b**) the NMAE of corrupted data.

**Figure 4:**
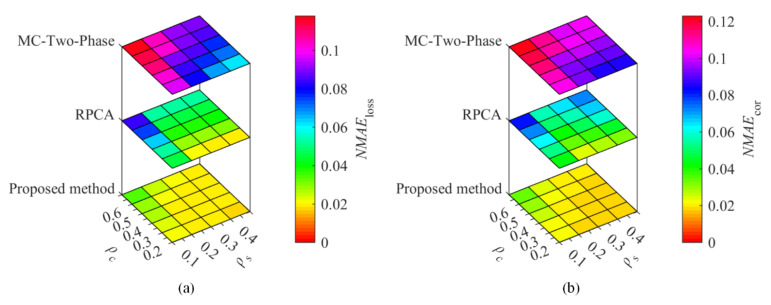
The recovery performance of each method for temperature data sensed in GreenOrbs. The (**a**) is the NMAE of missing data, (**b**) the NMAE of corrupted data.

**Figure 5:**
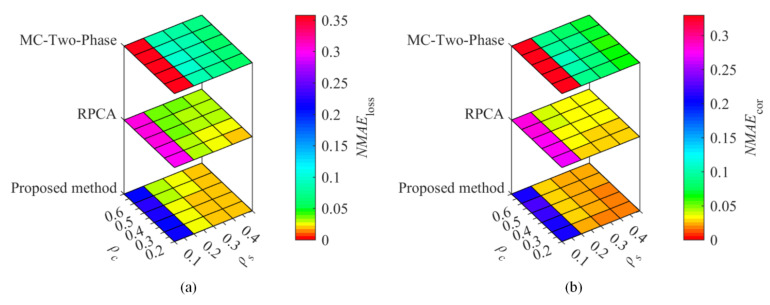
The recovery performance of each method for humidity data sensed in GreenOrbs. The (**a**) is the NMAE of missing data, (**b**) the NMAE of corrupted data.

To compare the proposed method with the existing methods, temperature and humidity data from the Inter Berkeley Research lab and GreenOrbs were utilized for the recovery performance comparisons. With the sampling ratio $\rho_{s}=$ 0.1, 0.2, 0.3, and 0.4, the corruption ratio $\rho_{c}=$ 0.2, 0.3, 0.4, 0.5, and 0.6, Figure 2 and Figure 3 show the recovery performance of each method for Berkeley temperature and humidity data, while Figure 4 and Figure 5 show the recovery performance of each method for GreenOrbs temperature and humidity data, respectively. As can be seen, the proposed method has a lower NMAE than the comparison methods on both missing and corrupted data in two datasets, especially at low sampling ratio and high corruption ratio.

As shown in Figure 2b, the $NMAE_{\mathrm{cor}}$ of the proposed method, RPCA, and MC-Two-Phase is 0.030, 0.037, and 0.099 when $\rho_{s}=0.2$ and $\rho_{c}=0.2$, while the values are 0.048, 0.097, and 0.140 when $\rho_{c}$ is up to 0.6. The results show that the proposed method not only improves the recovery accuracy of WSNs data but also has strong robustness to the gross noise.

Especially, from Figure 4, we can see that as the $\rho_{c}$ increases from 0.2 to 0.6, the corresponding $NMAE_{\mathrm{loss}}$ and $NMAE_{\mathrm{cor}}$ of MC-Two-Phase and RPCA dramatically increase, while that has very little change of the proposed method, and even the entire range of change is only less than 0.01.

As shown in Figure 3, the $NMAE_{\mathrm{loss}}$ and $NMAE_{\mathrm{cor}}$ of the proposed method decrease rapidly with the increase in $\rho_{s}$, but there is little change for the MC-Two-Phase. Specifically, in Figure 3b, the $NMAE_{\mathrm{cor}}$ of the proposed method, RPCA and MC-Two-Phase is 0.056, 0.069, and 0.076 when $\rho_{s}=0.1$ and $\rho_{c}=0.4$, while the values are 0.029, 0.040, and 0.069 when $\rho_{s}$ is up to 0.4.

## 5. Conclusions

In this paper, we propose a WRPCA method to increase the recovery accuracy of WSNs data with loss and corruption. The original data matrix is treated as a sum of a low-rank normal data matrix and a sparse abnormal matrix to avoid the influence of corruption. In addition, the weighted nuclear norm minimization is utilized to further constrain the low-rankness of the normal data and overcome the problem that the nuclear norm minimization treats all singular values equally. The experimental results show that the proposed method has better recovery performance in both loss and corruption data. In further work, the higher-order low-rankness of multi-attribute data in WSNs can be explored for multi-attribute data reconstruction.

## Data Availability

All data in this study are available upon request to the correspondence author.
